# Impact of Imaging Modality on AI-Based Detection of Incidental Maxillary Sinus Pathology: Comparison of Panoramic Radiography and CBCT

**DOI:** 10.3390/diagnostics16111667

**Published:** 2026-05-28

**Authors:** Anna Lackowska, Natalia Kazimierczak, Natalia Chwarścianek, Nora Sultani, Zbigniew Serafin, Wojciech Kazimierczak

**Affiliations:** 1 Department of Radiology and Diagnostic Imaging, Collegium Medicum, Nicolaus Copernicus University in Torun, Jagiellońska 13-15, 85-067 Bydgoszcz, Poland; 2 Kazimierczak Clinic, Dworcowa 13/u6a, 85-009 Bydgoszcz, Poland; 3 Independent Researcher, 87-100 Torun, Poland; 4 Faculty of Medicine, Collegium Medicum, Nicolaus Copernicus University in Torun, Jagiellońska 13-15, 85-067 Bydgoszcz, Poland; 5 Faculty of Medicine, Bydgoszcz University of Science and Technology, Kaliskiego 7, 85-796 Bydgoszcz, Poland

**Keywords:** artificial intelligence, cone-beam computed tomography, orthopantomogram, maxillary sinusitis, dental imaging, diagnostic accuracy, opportunistic screening, deep learning

## Abstract

**Background/Objectives:** The objective of our study was to compare the diagnostic performance of a popular, commercial dental artificial intelligence (AI) platform (Diagnocat, DGNCT LLC, Miami, FL, USA) for detecting maxillary sinus abnormalities on paired panoramic radiographs (OPG) and cone-beam computed tomography (CBCT) acquired in the same patients, and to examine whether lesion conspicuity predicts correct AI decisions. **Methods:** In this retrospective paired study, 166 patients contributed 332 maxillary sinuses with OPG and CBCT performed ≤30 days apart. The reference standard was consensus CBCT reading by two observers with third-reader arbitration. The index test was the AI’s sinus-level binary output (any abnormality). Accuracy, precision, recall, and F1 score were estimated with patient-clustered 95% bootstrap CIs; secondary analyses assessed category-specific performance and the effect of mucosal thickness and polyp/cyst volume. **Results:** Our evaluation showed that the platform’s performance depended on modality. On CBCT, the accuracy was 69.88% (64.76–74.70%), precision was 87.83% (81.58–93.33%), recall was 54.01% (46.74–61.17%), and F1 score was 66.89% (60.34–72.84%). On OPG, the accuracy was 50.6% (44.58–55.41%), precision was 67.80% (55.38–79.66%), recall was 21.39% (15.62–27.32%), and F1 score 32.52% (24.79–39.69%). On CBCT, higher mucosal thickness and larger polyp/cyst volume strongly predicted correct AI calls; no such effect was seen with OPG. **Conclusions:** In conclusion, the evaluated AI showed high precision but only moderate recall on CBCT and unreliable performance on OPG. Outputs must be interpreted by a professional; AI alerts on OPG should not guide management without CBCT confirmation.

## 1. Introduction

The incidence of maxillary sinusitis changes in cone-beam computed tomography (CBCT) varies depending on the study. According to Hsiao YJ et al. maxillary sinus pathology can be observed in 37.21% [1] CBCT examinations, while Dogan ME et al. [2] found pathology in 45.8% CBCT images. Among the sinus pathologies found on CBCT, the most common are mucosal thickening, mucosal polyp/mucosal retention cyst, and odontogenic sinusitis [3]. Dentists and oral radiologists are frequently the first healthcare professionals to identify these findings, placing them in a critical position for patient management and timely referral to otolaryngology specialists. The accurate detection of such incidental pathology is therefore of significant clinical importance.

An orthopantomogram (OPG) provides a broad overview of the masticatory system and the complete dentition; however, it is not specifically designed for detailed evaluation of the maxillary sinuses. Therefore, its accuracy in diagnosing radiological signs of sinusitis is limited. Unlike three-dimensional (3D) imaging, an OPG image has the inherent limitations of structures’ superimposition and geometric distortion [4,5,6]. As the study of Constantine et al. showed, the sensitivity of OPG for the detection of any maxillary sinus pathology was poor compared with CBCT, but the specificity was high [3].

The use of CBCT in dental practice is rising, as it provides detailed information on the root canal anatomy, periapical lesions, and adjacent anatomical structures [7,8]. A high-resolution 3D visualization of the oral and maxillofacial region can be crucial in better diagnosis and planning treatment, especially in procedures that take place proximal to the sinus floor, such as dental implant placement and sinus floor elevation [9]. Due to superior resolution, growing availability, and low radiation dose, the role of CBCT in maxillary sinus evaluation is constantly growing [10]. The present study is intentionally a real-world evaluation of a deployed clinical platform rather than a benchmark of all AI architectures.

Diagnocat is a dental artificial intelligence (AI) software using convolutional neural networks (CNNs), analyzing dental images (including 3D CBCTs and two-dimensional (2D) images like OPGs, bitewings, and periapical radiographs), identifying anomalies including dental caries, missing teeth, periapical lesions, and maxillary sinus abnormalities. According to the producer (DGNCT LLC, Miami, FL, USA), the information outlines the health of every tooth and can identify more than 40 different possible conditions on 2D images and more than 60 on 3D radiographs [11]. Diagnocat AI platform exhibited high diagnostic accuracy for evaluating endodontic treatment outcomes using CBCT [12,13] and significant potential on OPG [14,15,16] images. However, the detailed information regarding the system’s architecture, training, and hardware remains confidential.

While numerous studies [17,18,19] have evaluated AI performance for sinusitis detection on either OPG or CBCT in isolation, a critical knowledge gap exists. There is a lack of research directly comparing an AI’s performance across both modalities using a robust, paired-image design from the same patient cohort. Moreover, to our knowledge, no studies evaluating Diagnocat’s diagnostic accuracy in maxillary sinus evaluation have been published. This is a crucial omission, as clinicians investing in commercial AI platforms that claim to analyze both 2D and 3D images need evidence-based guidance on whether a tool’s performance is consistent across these fundamentally different data types. Without this evidence, the reliability of AI-generated alerts remains uncertain.

Therefore, the aim was to quantify, within the same patients, the difference in diagnostic performance of a commercial AI platform for detecting signs of maxillary sinusitis on OPG versus CBCT, using a consensus CBCT reference. We hypothesized that diagnostic accuracy would be significantly higher on CBCT than on OPG, and that lesion conspicuity (mucosal thickness; polyp/cyst volume) would positively predict correct AI classifications. We selected Diagnocat because it is widely deployed in our setting and supports both 2D and 3D dental imaging within a single clinical workflow.

## 2. Materials and Methods

### 2.1. Study Design

This retrospective diagnostic accuracy study was conducted in accordance with the ethical principles outlined in the Declaration of Helsinki. Ethical approval was obtained from the Ethics Committee of Collegium Medicum, Nicolaus Copernicus University in Torun, Poland (Protocol No. 274/2025, Date: 23 April 2025). Informed consent was waived due to the retrospective nature of the study and the anonymization of patient data. The study adheres to the Standards for Reporting of Diagnostic Accuracy Studies (STARD) 2015 guidelines [20].

### 2.2. Patients

The study population initially comprised 264 consecutive patients referred for OPG and CBCT imaging at a private dental center between January 2023 and March 2025. Referrals for both imaging modalities were made primarily by orthodontists or dental surgeons. All CBCT examinations with a large field of view (FOV) of 13 × 10 cm were clinically indicated (evaluation of periapical disease or impacted teeth with anticipated sinus proximity) and performed under ALARA principles consistent with European radioprotection guidance. No additional imaging was obtained for research purposes; analyses were retrospective, based on the anonymized data already in the record.

The primary inclusion criterion was the availability of both OPG and CBCT images for the same patient, acquired within a maximum interval of 30 days. This maximum interval of 30 days was deemed clinically acceptable to minimize the likelihood of significant pathological changes, such as the resolution of acute inflammation or the development of new pathology, occurring between scans, aligning with typical follow-up periods for non-acute sinonasal conditions.

Exclusion criteria included (1) incomplete coverage of the maxillary sinuses to the maxillary hiatus and dentition in either scan, (2) scans with severe motion artifacts compromising diagnostic quality, and (3) overall poor image quality.

### 2.3. Sample Size, Precision, and a Priori Power

The primary precision target was to achieve 95% confidence-interval (CI) half-widths ≤10 percentage points for the key diagnostic metrics (recall and accuracy) per modality. Using patient-clustered bootstrap (2000 replicates), the achieved half-widths at *n* = 166 patients/332 sinuses were 7.6 pp (CBCT recall), 5.0 pp (CBCT accuracy), 5.6 pp (OPG recall), and 5.6 pp (OPG accuracy), all meeting this criterion.

A power calculation for McNemar’s test was performed for the between-modality comparison (McNemar’s test for paired binary outcomes). Expected recall values were derived conservatively from the closest published study: Serindere et al. [21], who reported CNN sensitivity of 75.7% on panoramic radiographs and 100% on CBCT for maxillary sinusitis. Because Diagnocat is a general-purpose dental AI—not a sinus-specific model—we applied a conservative downward adjustment of approximately 35–40%, yielding prior estimates of CBCT recall ≈ 65%, and OPG recall ≈ 30%. With an anticipated disease prevalence of ~50% and assuming approximate independence of classification errors between modalities, the expected discordant pair probabilities were p_01_ = 0.228 (CBCT detects, OPG misses) and p_10_ = 0.053 (OPG detects, CBCT misses). Applying the method presented by Lachenbruch [22], the minimum required sample was *n* = 70 patient pairs. To support secondary subgroup analyses, a recruitment target of ≥150 patients was set. The enrolled cohort of *n* = 166 patients exceeds this requirement by 96 patients, yielding an achieved power of 98.9% at α = 0.05.

### 2.4. Image Acquisition

All OPG and CBCT images were acquired using a single Hyperion X9 PRO 13 × 16 unit (MyRay, Imola, Italy). Standardized acquisition parameters, designated as the “Regular” preset on the device, were used for all CBCT scans: 90 kV, 36 mAs, CTDI/Vol 4.09 mGy, and a 13 × 10 cm FOV. CBCT images were reconstructed with a 0.3 mm slice thickness. All patient identifiers were removed from the images prior to analysis to ensure anonymity, and each dataset was assigned a unique code for blinded evaluation.

### 2.5. AI Evaluation

Both OPG and CBCT image datasets in DICOM format, for each included patient, were manually uploaded to the Diagnocat cloud-based AI platform version 1.0 (DGNCT LLC, Miami, FL, USA) accessed on 10–20 April 2025. The software analyzed the maxillary sinuses for signs indicative of sinusitis. When abnormalities suggestive of sinusitis were detected, the platform automatically highlighted the affected areas in the reports generated for both OPG and CBCT modalities. The binary outcome (presence or absence of a finding) was recorded for each sinus (left and right), but the AI was not able to classify the lesion type. It was only able to trigger a general alert when the lesion was conspicuous enough. The AI’s assessment results regarding maxillary sinus findings were manually extracted from these reports and recorded in separate spreadsheets for OPG and CBCT data.

### 2.6. Human Observer Evaluation (Reference Standard)

CBCT images were independently evaluated by two observers: Observer 1, a radiology resident with 5 years of experience in maxillofacial imaging, and Observer 2, a dentist with 12 years of experience in dentomaxillofacial imaging. Readers were blinded to AI outputs and clinical data.

Images were reviewed on a medical-grade display (RadiForce MX243W) using iRYS Viewer software version 6.2 (from MyRay, Italy) in a dimmed reporting room. Given that CBCT lacks calibrated Hounsfield scaling, window/level values refer to vendor gray-level units; we standardized viewing to WW 4096/WL 2048 to utilize full dynamic range. Two blinded observers read independently; disagreements were resolved by a third board-certified radiologist (arbitration). The arbitrated or reader consensus served as the reference standard.

**Figure 1:**
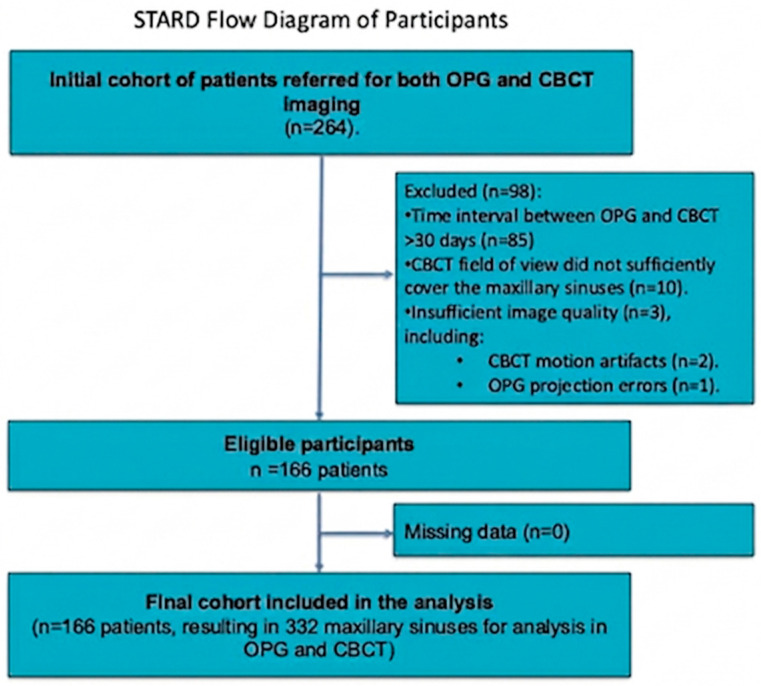
STARD 2015 flow diagram showing patient identification, eligibility, exclusions, and final cohort with per-sinus analysis.

Observers evaluated each maxillary sinus for the presence of abnormalities consistent with sinusitis, including mucosal thickening, cysts, polypoid lesions, and fluid levels (Figure 1). Mucosal thickening measuring 2 mm or greater in its maximum dimension was considered a positive finding, in accordance with established radiological criteria for distinguishing pathological thickening from normal physiological variation in the maxillary sinus mucosa [23,24,25,26,27].

When abnormalities were detected, the maximum cranio-caudal dimension of the thickened mucosa was measured. For polyps/retention cysts, volumes (mm^3^) were obtained using a simplified formula for oval lesions volumetry (ellipsoid method [28]):

V=A×B×C×0.52

where V is volume; A, B, C are orthogonal diameters (length, width, height) of lesions measured in coronal, axial, and sagittal planes; 0.52 is a rounded surrogate for π/6 (0.5236). This introduces <0.7% relative error compared with the exact factor. This method assumes an approximately ellipsoidal shape of polypoid lesions. Findings for each sinus were recorded in spreadsheets.

To assess reliability, inter-observer agreement was calculated for the initial readings. Additionally, intra-observer agreement was determined by having both readers re-evaluate 20% of the cases, randomly selected, one month after the initial assessment.

The consensus evaluation of CBCT images served as the definitive reference standard for assessing the AI’s performance on both CBCT and OPG modalities. This approach was chosen due to the established limitations and lower diagnostic sensitivity of OPG for maxillary sinus pathology compared to 3D imaging.

### 2.7. Statistical Evaluation

For diagnostic accuracy calculations, the reference standard alone defined disease status (positive if the consensus CBCT reading identified any abnormality ≥2 mm; negative otherwise). The index test (AI) was positive when the software flagged an abnormality and negative otherwise. While the AI platform provides a sinus-level, binary output indicating only the ‘presence or absence of any abnormality,’ our secondary analysis sought to understand the AI’s sensitivity to specific types of pathology as defined by the reference standard (e.g., mucosal thickening, polyps/cysts, free fluid).

To achieve this, we conducted category-specific performance analyses. For each specific finding, such as ‘polyp/retention cyst,’ the reference standard was used to define the ground truth (i.e., all sinuses with a consensus-confirmed polyp/cyst were considered ‘positive’ for that category). The AI’s binary ‘any abnormality’ output was then tested against this specific ground truth.

For example, in the polyp analysis,

• A true positive (TP) was recorded if the AI flagged ‘any abnormality’ in a sinus where the reference standard identified a polyp/cyst;
• A false negative (FN) was recorded if the AI did not flag ‘any abnormality’ in a sinus where the reference standard identified a polyp/cyst.

This approach allows for evaluating whether specific pathological signs, which are subsets of ‘any abnormality,’ are sufficient to trigger a positive finding from the AI. However, it is important to note that this method assesses the correlation between the presence of a specific pathology and the AI’s general alert; it does not confirm that the AI identified the lesion as a polyp, but rather that the polyp was conspicuous enough to be flagged as an abnormality.

To formally compare diagnostic performance between OPG and CBCT modalities, we performed McNemar’s test for paired proportions, the recommended approach for comparing two classifiers applied to the same subjects.

The diagnostic performance of the AI program was assessed by comparison to the readers’ consensus. The accuracy, precision, recall, and F1 score were calculated to assess AI results. Confidence intervals were calculated with the bootstrap method. Analyses were conducted per sinus with patient-level clustering (two sinuses/patient). We computed confusion matrices (TP, FP, TN, FN) and accuracy, precision, recall, and F1 score, each with 95% cluster-robust confidence intervals. Primary ‘any abnormality’ metrics were summarized with 95% patient-clustered bootstrap CIs (2000 resamples). For category-specific analyses (mucosal thickening, polyps/cysts, free fluid), we reported non-clustered 95% bootstrap CIs as pre-specified secondary endpoints. Primary comparisons were reported by modality (OPG vs. CBCT).

The means, standard deviations, medians, quartiles, and ranges of quantitative variables are shown. For qualitative variables, absolute and relative frequencies (N and %) are reported. The Mann–Whitney test was used for comparisons of quantitative variables between two groups. The cut point for the quantitative variable’s impact on the dichotomous variable was set based on the ROC curve. The point closest to the top-left corner was used. Inter-rater reliability of quantitative measures between raters was assessed with the intraclass correlation coefficient of type 2 (according to Shrout and Fleiss) [29]. Inter-rater reliability of qualitative measures between two raters was assessed with Cohen’s kappa [30]. In detailed analyses of the influence of the size or thickness of individual lesions on the correct Diagnocat diagnosis, only sites where the reader found abnormality were included. The formulas used in diagnostic performance calculations and further explanations can be found in Hicks et al. [31]. All the analyses were conducted in R software, version 4.5.1. Bootstrap confidence intervals were calculated using the ‘boot’ package in R, and ROC analysis was performed using the ‘pROC’ package.

## 3. Results

### 3.1. Patients

Following the initial screening, 85 patients were excluded due to an interval exceeding 30 days between the OPG and CBCT acquisitions. Further, 10 patient datasets were excluded because the CBCT field of view did not encompass the maxillary sinuses up to the level of the maxillary ostium. Three additional datasets were excluded due to insufficient image quality (CBCT motion artifacts, n = 2; OPG projection errors, n = 1). After applying all inclusion and exclusion criteria, a final cohort of 166 consecutive patients was included. Analyses were performed at the sinus level (per side), yielding a total of 332 maxillary sinuses from these patients (53 males and 113 females; mean age, 31.25 years; age range, 8–70 years) who were referred for both OPG and CBCT imaging. The prevalence of any abnormality by the reference standard was 55.7% (185/332 sinuses). The baseline patient and sinus characteristics are presented in Table 1. STARD flow diagram for the current study is presented in Figure 1. No missing data occurred; all AI analyses produced determinate outputs. No interim analyses were conducted. A complete STARD 2015 checklist is provided in Supplementary Table S1.

### 3.2. Diagnostic Accuracy Parameters

**Figure 2:**
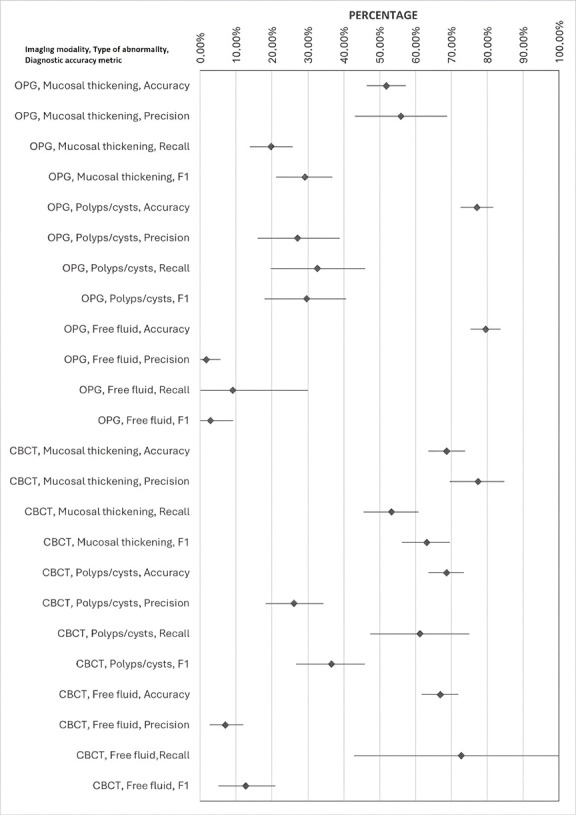
Diagnostic accuracy metrics of an AI platform for maxillary sinusitis (sinus level). Forest of an AI platform for maxillary sinusitis (sinus level). Bar plots display mean values of accuracy, precision, recall, and F1 score with 95% bootstrap confidence intervals (whiskers).

Table 2 presents the diagnostic performance of AI compared to reader evaluations across OPG and CBCT images. Artificial intelligence was more accurate in diagnosing any abnormalities characteristic of maxillary sinusitis on CBCT than on OPG (on CBCT, the accuracy was 69.9%, precision = 87.8%, recall = 54%, and the F1 score was 66.9%; on OPG, the accuracy was 50%, precision = 67.8%, recall = 21.4%, and the F1 score was 32.5%). The results platform’s diagnostic accuracy metrics are presented in Figure 2.

Although CBCT outperformed OPG overall, OPG showed numerically higher accuracy than CBCT for polyps/cysts and free fluid in our dataset. We attribute this to small numbers for free fluid and potential 2D over- or under-calling in specific categories; these findings are interpreted cautiously with CIs reported in the tables.

**Figure 3:**
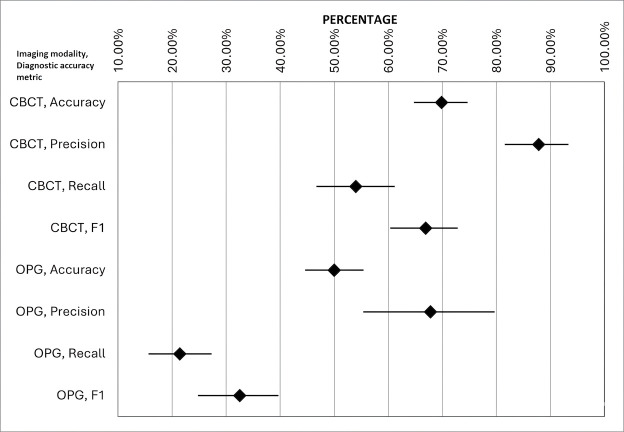
Diagnostic accuracy metrics for the detection of any maxillary sinus abnormality by AI on CBCT and OPG. Forrest plots show 95% confidence intervals computed with patient-level clustered bootstrap (N = 2000).

When analyzed on the patient (clustered) level, on CBCT, AI achieved high precision with a moderate F1 score driven by limited recall; on OPG, performance hovered near chance with very low recall, despite moderate precision. The results of diagnostic accuracy calculations on the patient level are similar to those on the sinus level (Table 3 and Figure 3).

### 3.3. Relationship Between Lesion Size and AI Platforms’ Diagnostic Accuracy on OPG and CBCT

Analyses of AI-based classifications of maxillary sinus lesions revealed a substantial number of misdiagnoses. In the subsequent phase, we examined the relationship between lesion size—defined as the median of the two observers’ measurements on CBCT images—and diagnostic outcomes (true positives versus false negatives). The analyses demonstrated a statistically significant association between lesion size and diagnostic correctness on CBCT evaluations (*p* < 0.001), whereas no statistically significant association was observed on OPG (*p* > 0.9). Detailed results are presented in further tables and figures.

The difference in volume of sinus polyps/cysts between correct and incorrect diagnoses of the Diagnocat software on OPG is statistically insignificant (Table 4). However, there is statistically significant (*p* < 0.05) polyp/cyst volume influence on correct Diagnocat diagnosis on CBCT.

**Figure 4:**
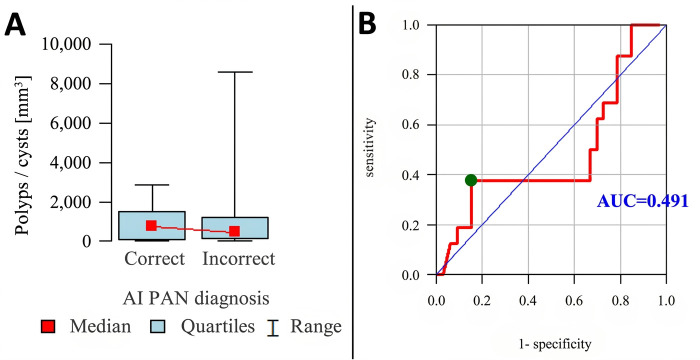
Effect of sinus polyp/cyst volume on Diagnocat diagnostic accuracy on OPGs. (**A**) Box-and-whisker plot—red point indicates the median; the box represents the interquartile range; whiskers indicate the full range. (**B**) ROC curve with the corresponding AUC.

**Figure 5:**
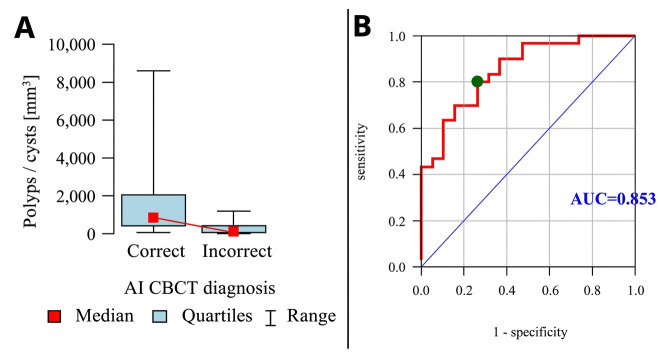
Effect of sinus polyp/cyst volume on Diagnocat diagnostic accuracy on CBCT. (**A**) Box-and-whisker plot—red point indicates the median; the box represents the interquartile range; whiskers indicate the full range. (**B**) ROC curve with the corresponding AUC.

As shown in Figure 4, polyp/retention cyst volume is a poor predictor for a correct diagnosis on the OPG, as evidenced by the receiver operating characteristic (ROC) curve with the corresponding area under the curve (AUC) value of 0.491. However, polyp/retention cyst volume is a very good predictor for a correct AI diagnosis on CBCT (ROC-AUC value: 0.853). When the polyp/retention cyst volume is above 400.4 mm^3^, a correct AI diagnosis on CBCT should be expected (sensitivity of 80.0% and specificity of 73.7%) (Figure 5).

The difference in mucosal thickness between correct and incorrect diagnoses of the Diagnocat software on OPG is statistically insignificant (Table 5). There is a statistically significant (*p* < 0.05) mucosal thickness influence on correct Diagnocat diagnosis on CBCT.

**Figure 6:**
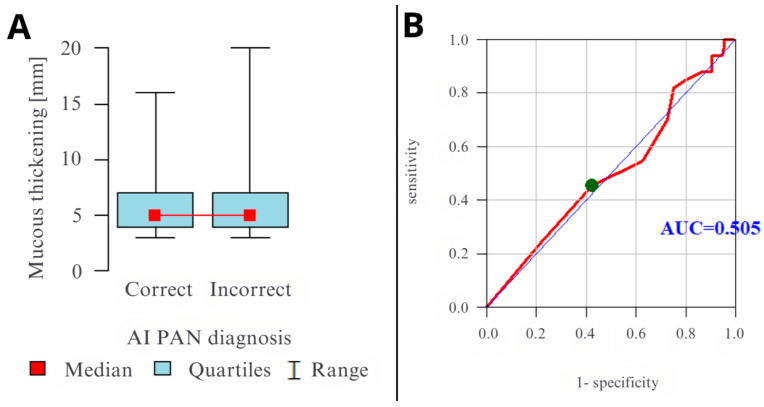
Graphs presenting the influence of mucosal thickness on the correct Diagnocat diagnosis on OPG. (**A**) Box-and-whisker plot—red point indicates the median; the box represents the interquartile range; whiskers indicate the full range. (**B**) ROC curve with the corresponding AUC.

**Figure 7:**
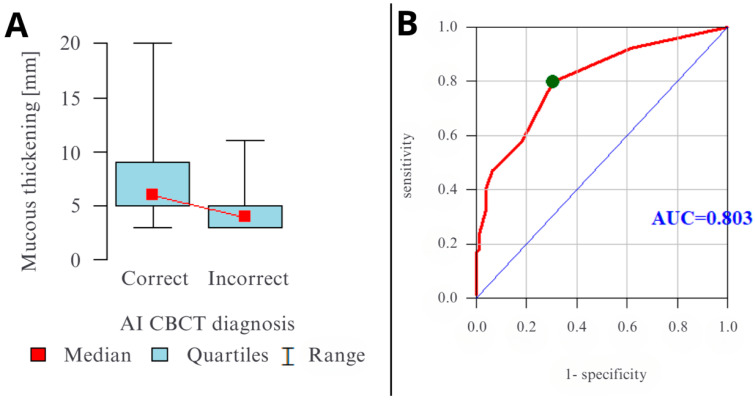
Graphs presenting the influence of mucosal thickness on the correct Diagnocat diagnosis on CBCT. (**A**) Box-and-whisker plot—red point indicates the median; the box represents the interquartile range; whiskers indicate the full range. (**B**) ROC curve with the corresponding AUC.

As shown in Figure 6, mucosal thickness is a poor predictor for a correct diagnosis on OPG, as evidenced by the ROC (AUC) value of 0.505. Mucosal thickness is a very good predictor for a correct AI diagnosis on CBCT, with the ROC (AUC) value of 0.803 (Figure 7). When mucosal thickness is greater than 5 mm, a correct AI diagnosis on CBCT should be expected (sensitivity of 79.8% and a specificity of 69.2%).

### 3.4. Formal Paired Statistical Comparison

To directly compare the diagnostic performance of the AI platform across modalities, we performed McNemar’s test for paired proportions. When comparing the overall binary AI outputs (presence/absence of abnormality), McNemar’s test revealed a highly significant difference between OPG and CBCT (χ^2^ = 16.06, *p* < 0.001). This difference was driven by asymmetric discordant pairs: of the 72 cases where OPG and CBCT disagreed, 53 cases (73.6%) had CBCT positive and OPG negative, whereas only 19 cases (26.4%) had OPG positive and CBCT negative (Table 6).

Among the 120 abnormal reference cases, McNemar’s test confirmed significantly higher recall for CBCT (76/120, 63.33%) compared to OPG (43/120, 35.83%), representing an absolute difference of 27.50 percentage points (χ^2^ = 25.88, *p* < 0.001). Of the 57 discordant abnormal cases, CBCT detected the abnormality in 45 cases (78.9%), while OPG detected it in only 12 cases (21.1%), indicating CBCT’s substantially higher recall. The results of McNemar’s tests on paired AI diagnostic accuracy are presented in Table 7.

### 3.5. Inter- and Intra-Reader Agreement

Inter-reader agreement was substantial to almost perfect (e.g., κ = 0.813 for “any abnormality”), and repeated-measure reliability was excellent (ICC ≈ 0.91). Full estimates and CIs are provided in Supplementary Tables S2 and S3.

## 4. Discussion

The central finding of this study is that the diagnostic performance of a commercial, generalist dental AI for detecting maxillary sinus pathology is fundamentally dependent on imaging modality. The AI demonstrated moderate utility on three-dimensional CBCT but was unreliable on two-dimensional OPGs, where its performance was equivalent to chance. However, even on CBCT, the AI’s recall was moderate (≈54%), indicating that subtle lesions are frequently missed despite high precision. Even on CBCT, the AI missed approximately 46% of reference-standard positive sinuses. Therefore, a negative AI result cannot be used to exclude maxillary sinus pathology or to reassure the clinician in patients with symptoms, dental infection, implant-planning indications, or radiological suspicion. The system may be useful as an adjunctive positive-alert tool for conspicuous lesions, but it is not suitable as a stand-alone screening or rule-out instrument.

The recent systematic review of 12 studies with a total of 3349 patients (7358 images), the performance of AI in detecting maxillary sinus pathology on CT and CBCT varied, with accuracy ranging from 85% to 97% and sensitivity ranging from 87% to 100% [32]. In our study, the results (accuracy = 69.9% and sensitivity = 54%) are weaker compared to the above systematic review. This observed performance is likely a characteristic feature of a “generalist” AI platform rather than an anomaly. The evaluated software is marketed as a comprehensive tool capable of identifying over 60 different conditions on 3D radiographs. Such a broad-spectrum model cannot be expected to match the performance of highly specialized, single-task research models, which consistently report accuracies exceeding 85% for maxillary sinusitis detection [21]. The modest accuracy on CBCT (69.9%) found in our study is therefore an expected outcome of the trade-off between an AI’s breadth of application and its depth of performance on any single, non-primary task. This provides a crucial insight for clinicians evaluating “all-in-one” AI solutions.

A study by Serindere et al. [21] evaluated the diagnostic accuracy of a convolutional neural network (CNN) model in diagnosing maxillary sinusitis on OPGs and CBCTs. The model was trained and tested by applying fivefold cross-validation to a dataset of 148 healthy and 148 inflamed sinus images. Comparable to our study, the diagnostic performance of the CNN for maxillary sinusitis was clearly higher with CBCT images; however, both OPG and CBCT accuracy and sensitivity were higher than in our study (for OPG, 75.7% for both accuracy and sensitivity, and for CBCT, 99.7% and 100%, respectively). The difference in AI diagnostic accuracy between 2D and 3D images is not a surprising result, as CBCT provides detailed 3D imaging for precise assessment and diagnosis of sinus-related issues. The recent studies showed that Diagnocat exhibited significantly higher sensitivity in assessment for endodontic treatment outcomes and periapical lesions detection on CBCT than on OPG [13,15,33].

The dramatic performance gap between modalities can be explained from an imaging physics perspective. The AI’s failure on OPG is not necessarily a flaw in the algorithm but a reflection of the profound limitations of the input data. The superimposition of dense anatomical structures (such as the zygomatic process and hard palate) on a 2D image inherently reduces lesion conspicuity to a level that even advanced pattern recognition algorithms cannot reliably overcome, as OPG imaging has low efficacy in the diagnosis of sinus disease, even when examined by experienced dental radiologists. However, it can be useful in excluding the disease [3]. Kuwana et al. showed an impressive over 89% sensitivity of their AI model in object detection in the diagnosis of maxillary sinuses on OPGs [34]. However, their dataset presented typical, preselected images depicting healthy sinuses, inflamed maxillary sinuses, and cysts of the maxillary sinus region. Our datasets, comprising consecutive patients presenting to a private dental practice, are more representative of real-world clinical settings, where patients often exhibit heterogeneous, non-textbook manifestations of disease.

Our results indicate that mucosal thickening and polyp/cyst volume are strong predictors of a correct Diagnocat diagnosis on CBCT. Furthermore, both polyp/cyst volume and the degree of mucosal thickening were significantly greater when the AI–CBCT diagnosis was correct than when it was incorrect. Among CBCT findings characteristic of maxillary sinusitis, Diagnocat achieved the highest precision and accuracy in detecting mucosal thickening. The AI’s success was strongly correlated with lesion size, as evidenced by the high AUC values. This suggests the AI functions primarily as a “conspicuity detector,” linking its behavior back to fundamental principles of medical imaging.

While CT scans are considered the “gold standard” for diagnosing sinusitis, particularly for dental planning, CBCT offers visualization of high-contrast morphology in the sinus, maxillofacial region, and otologic applications [35]. Moreover, radiation doses are comparable to or less than those reported for conventional diagnostic CT of the head [36]. CBCT is especially valuable for odontogenic (tooth-related) sinusitis [37,38] and provides detailed 3D views for dental and skeletal issues. However, CBCT usually only shows maxillary sinuses, and the remaining (frontal, ethmoid, sphenoid) sinuses can be seen in a dedicated CT.

These findings are consistent with the broader literature on the evaluated platform. The low sensitivity observed for sinus pathology on 2D images aligns with reports of its low sensitivity for other subtle pathologies, such as incipient caries and small periapical lesions [14,39]. This demonstrates a consistent performance pattern, reinforcing the “generalist AI” paradigm and suggesting its utility is greatest for more obvious findings on high-quality 3D imaging.

This study has several limitations. First, AI outputs available for this re-analysis were binary at the sinus level for “any abnormality” only; category-specific AI outputs (mucosal thickening, polyp/cyst, free fluid) were not present, precluding stratified performance estimates. Its retrospective, single-center design may introduce selection bias, as the patient cohort may not be representative of the general population. The use of a single commercial AI platform and a single CBCT scanner model means the results may not be generalizable to other AI systems or imaging hardware. Furthermore, the developer’s lack of transparency regarding the system’s architecture renders the platform effectively a “black box” from the perspective of commercial users. The 30-day interval between OPG and CBCT acquisitions represents a potential source of bias. Although this window is clinically pragmatic, transient physiological variations in the maxillary sinus mucosa—driven by allergic or infectious episodes—could influence the comparative analysis. A significant limitation of this study is the presence of reference standard bias. By using CBCT as the ‘ground truth’ for both modalities, the OPG arm is evaluated against a standard that captures findings beyond the physical diagnostic yield of 2D imaging. This creates a ceiling effect for OPG performance: the AI may be penalized for ‘failing’ to detect pathology that is simply not present in a 2D projection. Readers should interpret the performance gap between OPG and CBCT as an expression of both AI capability and the inherent physical limitations of the OPG modality itself. Finally, the reference standard was based on radiological interpretation alone; the ultimate ground truth of histopathological confirmation was not available, a common limitation in imaging research.

## 5. Conclusions

In conclusion, while AI holds considerable promise for opportunistic screening in dental radiology, our findings underscore that an algorithm’s performance is inextricably linked to the quality and dimensionality of the input data. The paired formal comparisons confirm that AI diagnostic performance is significantly modality-dependent, with CBCT providing superior sensitivity for abnormality detection. CBCT outputs are precise but miss a proportion of subtle lesions—a negative result on this opportunistic screening tool does not rule out every sinus pathology, and clinical correlation remains mandatory. OPG outputs are unreliable. Professional review remains essential, and OPG-based AI alerts should not guide management without CBCT confirmation.

## Figures and Tables

**Table 1:** Baseline patient and sinus characteristics.

Characteristic	Value
Patients	
Total number	166
Age, years (mean ± SD)	31.3 ± 15.2
Age, years (range)	8–70
Sex	
Female, n (%)	113 (68.1)
Male, n (%)	53 (31.9)
Sinuses	
Total number	332
Prevalence of Findings by Reference Standard (CBCT Consensus)	
Any abnormality, n (%)	185 (55.7)
Mucosal thickening (≥2 mm), n (%)	167 (50.3)
Polyp/Retention cyst, n (%)	49 (15.2)
Free fluid, n (%)	11 (3.4)
Lesion Characteristics (in positive cases)	
Mucosal thickening, mm (mean ± SD)	6.0 ± 3.7
Polyp/Cyst volume, mm^3^ (median)	463.3 [131.0–1216.8]

**Table 2:** Reference-standard category-specific conspicuity analysis of the AI’s binary “any abnormality” output.

Parameter	Modality	Accuracy	Precision	Recall	F1 Score
Mucosal thickening	OPG	51.81% (46.39–57.23%)	55.93% (43.14–68.75%)	19.76% (13.82–25.84%)	29.20% (21.20–36.75%)
Polyps/cysts		77.11% (72.59–81.63%)	27.12% (16.00–38.89%)	32.65% (19.65–46.00%)	29.63% (18.02–40.65%)
Free fluid		79.52% (75.30–83.73%)	1.69% (0.00–5.66%)	9.09% (0.00–30.00%)	2.86% (0.00–9.23%)
Any abnormalities		50.60% (44.58–55.41%)	67.80% (55.38–79.66%)	21.39% (15.62–27.32%)	32.52% (24.79–39.69%)
Mucosal thickening	CBCT	68.67% (63.55–73.80%)	77.39% (69.45–84.75%)	53.29% (45.51–60.87%)	63.12% (56.18–69.54%)
Polyps/cysts		68.67% (63.55–73.49%)	26.09% (18.18–34.31%)	61.22% (47.37–75.00%)	36.59% (26.75–45.81%)
Free fluid		66.87% (61.75–71.98%)	6.96% (2.65–12.00%)	72.73% (42.86–100.00%)	12.70% (5.04–20.90%)
Any abnormalities		69.88% (64.76–74.70%)	87.83% (81.58–93.33%)	54.01% (46.74–61.17%)	66.89% (60.34–72.84%)

Non-clustered 95% bootstrap confidence intervals are mentioned in parentheses. Category-specific rows do not represent AI classification of lesion type. They indicate whether sinuses with a given reference-standard finding triggered the AI’s general abnormality alert.

**Table 3:** Confusion matrix and diagnostic accuracy metrics of the AI platform for maxillary sinusitis assessment on OPG and CBCT at the sinus level (any abnormality, clustered by patient).

Modality	TP	FP	TN	FN	Accuracy	Precision	Recall	F1 Score
CBCT	100	15	132	85	69.9% (64.8–74.7%)	87.8% (81.6–93.3%)	54.0% (46.7–61.2%)	66.9% (60.3–72.8%)
OPG	40	19	128	145	50.6% (44.6–55.4%)	67.8% (55.4–79.7%)	21.4% (15.6–27.3%)	32.5% (24.8–39.7%)

TP—true positive; FP—false positive; TN—true negative; FN—false negative. Values in parentheses indicate 95% bootstrap confidence intervals.

**Table 4:** The influence of sinus polyp/cyst volume on the correct Diagnocat diagnosis on OPG and CBCT.

AI Diagnosis	Modality	N	Polyps/Cysts [mm^3^]							*p*
			Mean	SD	Median	Min	Max	Q1	Q3	
Correct	OPG	16	924.79	909.24	759.98	46.80	2845.44	109.20	1487.33	*p* = 0.924
Incorrect		33	1355.91	2278.70	463.32	24.96	8592.48	131.04	1216.80	
Correct	CBCT	30	1808.30	2284.67	837.98	62.40	8592.48	446.16	2010.97	*p* < 0.001 *
Incorrect		19	278.56	330.79	109.20	24.96	1216.80	71.76	436.28	

*p*—Mann–Whitney test, SD—standard deviation, Q1—lower quartile, Q3—upper quartile. * statistically significant (*p* < 0.05).

**Table 5:** The influence of mucosal thickness on the correct Diagnocat diagnosis on OPG and CBCT.

AI Diagnosis	Modality	N	Mucosal Thickening [mm]							*p*
			Mean	SD	Median	Min	Max	Q1	Q3	
Correct	OPG	33	5.91	3.31	5	3	16	4	7	*p* = 0.933
Incorrect		134	6.13	3.75	5	3	20	4	7	
Correct	CBCT	89	7.67	4.20	6	3	20	5	9	*p* < 0.001 *
Incorrect		78	4.27	1.56	4	3	11	3	5	

*p*—Mann–Whitney test, SD—standard deviation, Q1—lower quartile, Q3—upper quartile. * statistically significant (*p* < 0.05).

**Table 6:** Diagnostic performance metrics with 95% confidence intervals.

Metric	Modality	Count/Total	Value (%)	95% CI Lower	95% CI Upper	95% CI
Accuracy	OPG	77/166	46.39	38.97	53.97	38.97–53.97
	CBCT	109/166	65.66	58.16	72.46	58.16–72.46
Recall	OPG	43/120	35.83	27.82	44.73	27.82–44.73
	CBCT	76/120	63.33	54.42	71.42	54.42–71.42
Precision	OPG	43/55	78.18	65.63	87.05	65.63–87.05
	CBCT	76/89	85.39	76.60	91.26	76.60–91.26

**Table 7:** Performance comparison with 95% confidence intervals.

Metric	OPG (95% CI)	CBCT (95% CI)	Difference (pp)
Accuracy	46.39% (38.97–53.97%)	65.66% (58.16–72.46%)	+19.28
Recall	35.83% (27.82–44.73%)	63.33% (54.42–71.42%)	+27.50
Precision	78.18% (65.63–87.05%)	85.39% (76.60–91.26%)	+7.21
F1 Score	0.4914	0.7273	+0.2359

## Data Availability

The de-identified analysis dataset supporting the findings of this study (per-patient tables containing ground-truth labels, AI outputs, and derived variables) is uploaded as Supplementary Table S4. Due to legal/contractual constraints and patient privacy, raw DICOM imaging data cannot be shared publicly. Requests for controlled access to the raw images for verification purposes will be considered by the corresponding author and the institutional data custodian under a data use agreement that prohibits re-identification and onward sharing. De-identified per-sinus datasets are available from the corresponding author on reasonable request.

## Supplementary Materials

The following are available online at https://www.mdpi.com/article/10.3390/diagnostics16111667/s1, Table S1: A complete STARD 2015 checklist, Table S2: Sinus pathology measurement agreement between two readers. Table S3: Sinus pathology diagnoses agreement between two readers, Table S4: Analysis dataset Sinuses AI results and Consesnus spreadsheet.
