# Supplementary material for: Impact of Imaging Modality on AI-Based Detection of Incidental Maxillary Sinus Pathology: Comparison of Panoramic Radiography and CBCT
Source: Diagnostics (Basel). 2026 May 28;16(11):1667. doi: 10.3390/diagnostics16111667 (PMC13257295; doi:10.3390/diagnostics16111667)
Supplement: Supplementary file 1 [file diagnostics-16-01667-s001.zip › Supplementary Tables S2 and S3.pdf]

Supplementary Table S2. Sinus pathology measurement agreement between two readers.

Parameter	ICC	95% CI		Agreement (Koo & Li)
Mucosal thickening [mm]	0.900	0.877	0.918	Good
Polyps / cysts [mm^3]	0.909	0.867	0.959	Excellent

Supplementary Table S3. Sinus pathology diagnoses agreement between two readers.

Parameter	Agreement	κ	95% CI		Interpretation (Landis & Koch)
Mucosal thickening	87.88%	0.722	0.544	0.900	Substantial
Polyps / cysts	98.48%	0.915	0.749	1.000	Almost perfect
Any abnormalities	90.91%	0.813	0.671	0.954	Almost perfect
